# Diabetes-Related Patient Outcomes through Comprehensive Medication Management Delivered by Clinical Pharmacists in a Rural Family Medicine Clinic

**DOI:** 10.3390/pharmacy8030115

**Published:** 2020-07-09

**Authors:** Jarred Prudencio, Michelle Kim

**Affiliations:** Department of Pharmacy Practice, The Daniel K. Inouye College of Pharmacy, University of Hawaii at Hilo, Hilo, HI 96720, USA; msk@hawaii.edu

**Keywords:** diabetes, hypertension, dyslipidemia, primary care, family medicine, comprehensive medication management

## Abstract

Two clinical pharmacy faculty members from a college of pharmacy provide comprehensive medication management in a rural family medicine clinic. The data was assessed for patients with diabetes managed by the pharmacists from 1 January 2017 through to 31 December 2019 to determine the service’s impact on patient outcomes. The primary outcome of this study is the change in the goal attainment rates of the three clinical goals of hemoglobin A1c, blood pressure, and appropriate statin therapy after pharmacist intervention. A total of 207 patients were included. At baseline, the patients had an average of 1.13 of the three goals met, improving to an average of 2.02 goals met after pharmacist intervention (*p* < 0.001). At baseline, 4.8% of the patients had met all three clinical goals, improving to 30.9% after pharmacist intervention (*p* < 0.001). There were significant improvements for the individual goal attainment rates of hemoglobin A1c (24.15% vs. 51.21%, *p* < 0.001), blood pressure (42.51% vs. 85.51%, *p* < 0.001), and appropriate statin therapy (45.89% vs. 65.70%, *p* < 0.001). This data adds to the evidence supporting the integration of clinical pharmacists into primary care clinics to improve patient outcomes related to diabetes.

## 1. Introduction

Ambulatory care pharmacy has been a growing area of the clinical pharmacy profession, where pharmacists work with patients in the outpatient setting to ensure safe and effective medication utilization [1]. Although this is becoming a more common area of practice for pharmacists, there has not been a standardization of ambulatory care pharmacy services. The practice models can vary vastly among different clinical sites due to differences in business models, state laws and regulations, and varying degrees of interdisciplinary integration. Ambulatory care pharmacy services can be implemented in a variety of practice settings, including primary care, specialty care, or telehealth clinics. While there is a plethora of evidence that supports ambulatory care pharmacy in each of these settings, the benefit of a clinical pharmacist integrated into a primary care clinic is particularly well documented [2,3,4,5,6]. Although there are varying practices, comprehensive medication management (CMM) is becoming a prominent model for pharmacists embedded in primary care clinics [7]. CMM is a model of service provided by clinical pharmacists that ensures each patient’s medication regimen is optimized to ensure the highest safety and efficacy outcomes can be achieved, taking into account patient-specific factors [7].

The Daniel K. Inouye College of Pharmacy (DKICP) at the University of Hawaii at Hilo was established in 2007. The East Hawaii Health Clinic opened in 2009 as a primary care teaching clinic created to educate the future generations of healthcare workers. The clinic includes physicians, nurse practitioners, behavioral health specialists, nurses, and clinical pharmacist faculty members from the DKICP. The learners at this clinic include family medicine physician residents; clinical psychology fellows; and medical, nursing, and pharmacy students. The collaboration between the college and the clinic serves a dual purpose of providing interprofessional patient-centered care and education. As the clinic evolved, so did the clinical pharmacy service. Currently, there are two clinical pharmacist faculty members who have been at this clinic since August 2016 and have established a CMM service. Each pharmacist spends 3 days per week in the clinic and has their own panel of patients to manage, collectively representing 1.2 full-time equivalent (FTE) of pharmacist services. The pharmacists are funded by the DKICP as faculty. The pharmacist faculty precept pharmacy students and educate medical residents on pharmacotherapy topics in the didactic setting and through case consultations.

A common area of focus for pharmacists embedded into primary care clinics is working with patients on the management of diabetes and chronic cardiovascular conditions. Patients with diabetes are at a higher risk of cardiovascular complications, and managing diabetes includes a multitude of factors [8]. Many clinicians focus on three primary goals for patients with diabetes, which are hemoglobin A1c, blood pressure, and cholesterol, otherwise known as “the ABCs of Diabetes” [8]. The American Diabetes Association (ADA) recommends a hemoglobin A1c goal for the average patient with diabetes to be <7%, but a more stringent goal of <6.5% or a less stringent goal of <8% are often considered, depending on patient-specific factors [9]. The blood pressure goal of <140/90 mmHg is also commonly utilized for patients with diabetes [9,10]. While some organizations recommend a blood pressure goal of <130/80 mmHg, the ADA and the Eighth Joint National Committee (JNC8) recommended goal of <140/90 mmHg was chosen for this study [9,10]. Although cholesterol is a concern for patients with diabetes, current clinical practice guidelines focus primarily on utilizing a moderate-to-high intensity statin for patients with diabetes, as opposed to specific lipid panel goals [9,11]. When providing CMM for patients with diabetes, clinicians focus on ensuring that the patient meets these three clinical goals in order to achieve adequate chronic disease state control and prevent future complications. The goal of this study is to assess the impact that a clinical pharmacist-led CMM service has on outcomes for patients with diabetes, as evidenced by changes in the goal attainment rates for hemoglobin A1c, blood pressure, and appropriate statin therapy before and after the pharmacist intervention.

## 2. Materials and Methods

This study was conducted as a retrospective chart review and was approved by the University of Hawaii Institutional Review Board (Approval Protocol #2018-00938). The electronic medical records (EMR) were reviewed for patients who had at least one appointment with a clinical pharmacist between 1 January 2017 and 31 December 2019.

Patients were scheduled for CMM appointments with the clinical pharmacists through two different avenues. First, the patients could be referred by their primary care provider (PCP). Anecdotally, the majority of patient appointments were scheduled through this route. The majority of referrals from PCPs to the CMM service were due to uncontrolled diabetes, medication nonadherence, or a need for medication education and counseling. Second, patients could be identified by the clinical pharmacists through EMR review due to uncontrolled chronic conditions or potential polypharmacy issues. EMR review was primarily conducted in the first few months of the start of the service, and as the PCPs gained familiarity with the service, the referrals increased over time and less time was spent doing EMR review outreach. In both instances, the patient was then scheduled by the clerical staff and added onto the pharmacist’s panel of patients for an in-person visit.

The pharmacist appointments were all 40 min in duration and were conducted as in-person visits to the clinic. While each appointment may not take the full 40 min, this time was set to allow for an in-depth discussion between the pharmacist and the patient regarding their chronic conditions, medications, and lifestyle. This time also allowed for the incorporation of pharmacy student learners to participate in patient appointments. A comprehensive medication reconciliation was completed at the start of each visit to clarify any medication discrepancies or nonadherence. The pharmacist then spends the remainder of the visit providing motivational interviewing, medication, and lifestyle counseling, and clarifying any questions the patient may have. Both pharmacists have a progressive collaborative practice agreement (CPA) with all the physicians in this clinic. The CPA was created by the pharmacist faculty in collaboration with the clinic’s medical director. There is no specific credentialing or privileging process included in the CPA, as it is specific to the two DKICP faculty members, who are vetted by the clinic’s medical director and administration team during the hiring process. The CPA allows the pharmacist to make changes to the patient’s medication regimen including initiating, adjusting, or discontinuing any non-controlled prescription medications. The CPA does not include specific medications, circumstances, or treatment protocols that the pharmacist must follow. Instead, the CPA is openly worded to allow the pharmacist to select drug changes based on their own clinical judgement and knowledge of evidence-based medicine. The CPA also allows the pharmacist to order any relevant laboratory tests that the patient may need and is able to renew prescriptions that are needed. After making the necessary adjustments and providing education to the patient, the patient is then scheduled for a future follow-up appointment with the pharmacist or the PCP based on the discretion of the pharmacist. In both scenarios, the pharmacist is responsible for following up on any results from laboratory tests they order, whether that is discussing results with the patients directly or communicating with the PCP to relay that information. Patients are continuously managed by the pharmacist until their medication regimens remain stable and there is no immediate need for follow-up CMM appointments. At that point, the patients will follow up with their PCP for general wellness appointments and can be rescheduled for a CMM appointment with the pharmacist should the need arise again in the future.

Although the clinical pharmacy service truly is focused on comprehensive medication management as opposed to disease-specific management, the majority of patients referred to the CMM service have been for diabetes management. This is likely due to these pharmacists’ specific expertise in diabetes management and because a large portion of diabetes management is based on pharmacotherapy [9]. This clinic is located in a rural city, and patients do not readily have access to endocrinologists or dieticians, which adds to the reasons why the majority of patient referrals are for diabetes. For each of these patients, the 3 primary goals set are attaining a controlled hemoglobin A1c level (patient-specific but typically <7% or <8%), a blood pressure of <140/90 mmHg, and a prescription of a moderate-to-high intensity statin [9,10,11]. Each goal is set by the clinical pharmacist depending on the specific patient.

To be included in the study analysis, patients must have had a diagnosis of type 1 or type 2 diabetes and have had at least 1 appointment with a pharmacist for CMM. The patients were further excluded from the analysis if they were under 18 years of age or did not have an updated hemoglobin A1c or blood pressure reading after their CMM visit. The primary outcome of this study was the composite of goal attainment rates for patients with diabetes, measured as pre-pharmacist intervention (baseline) and post-pharmacist intervention. The additional secondary outcomes of this study include specific goal attainment rates for hemoglobin A1c, blood pressure, and statin prescription. The blood pressure and hemoglobin A1c values from the initial visit were documented as the baseline value, and the blood pressure and hemoglobin A1c values at the last pharmacist visit were documented as the post-pharmacist intervention value. Other included outcomes are changes in the average hemoglobin A1c and blood pressure, in addition to changes in the number and types of medications used to manage diabetes, hypertension, and dyslipidemia. Statistical analyses were conducted using paired t-tests and McNemar tests and conducted as a per protocol analysis. 

## 3. Results

Over the three-year period, there were a total of 1600 CMM visits with 337 patients managed by the clinical pharmacists between 1 January 2017 and 31 December 2019. Of the 337 patients seen by the CMM service, 224 (66.5%) had a diagnosis of diabetes. These 224 patients had a total of 1417 visits, representing 88.5% of the CMM visits. Of the 224, 17 patients were excluded, leaving a total of 207 patients to be included in the study analysis. Of the 17 excluded patients, 2 patients were under the age of 18 years, and 15 patients did not have an updated hemoglobin A1c after their CMM visit. 

Prior to the patients receiving any pharmacy interventions, 10 (4.8%) patients were able to attain all three of the primary clinical goals (a patient-specific controlled hemoglobin A1c level of <7% or <8%, a blood pressure of <140/90 mmHg, and a prescription of a moderate-to-high intensity statin). There were 43 (20.8%) patients that had not met any of the three goals at baseline, and about half (50.7%) of the patients had met one of the three goals. The most common goal attained at baseline was being prescribed an appropriate statin (45.89%). The full baseline characteristics can be found in Table 1.

**Figure 1:**
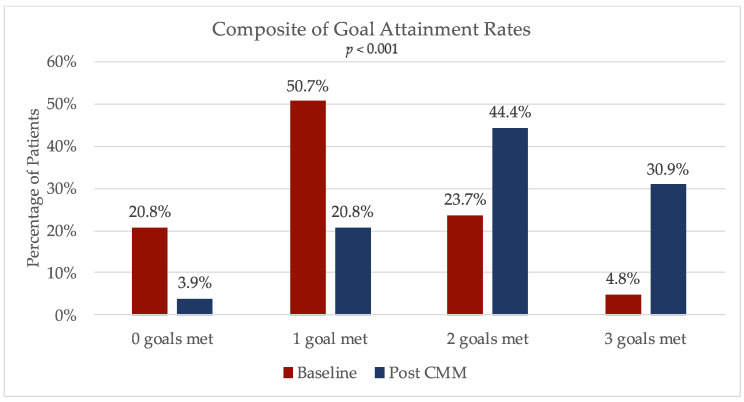
Primary outcome: change in composite of goal attainment rates with comprehensive medication management (CMM).

There was a significant increase in the composite of goal attainment rates after the clinical pharmacy interventions, representing the primary objective of the study as depicted in Figure 1 (*p* < 0.001). A total of 64 (30.9%) patients had met all three primary goals after the CMM visits, for an increase of 26.1 percentage points. Overall, 96.1% of all the patients had met at least one of the primary study goals after working with the pharmacist. At baseline, the average amount of goals met was 1.13, and this increased to an average of 2.02 after the CMM (*p* < 0.001).

**Figure 2:**
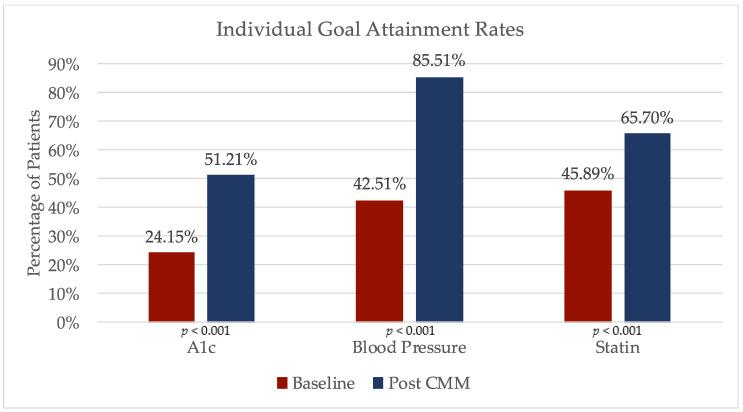
Secondary outcomes: change in individual goal attainment rates with CMM.

The secondary objectives of individual goal attainment rates of hemoglobin A1c, hypertension, and appropriate statin prescription were all found to be significantly improved after pharmacy intervention, as depicted in Figure 2. Prior to any pharmacy appointments, 50 (24.15%) patients had a controlled hemoglobin A1c at baseline but after the clinical pharmacy interventions, and 106 patients (51.21%) had reached their A1c goal, for an improvement of 27.06 percentage points (*p* < 0.001). At baseline, 88 (45.21%) patients had a blood pressure considered to be controlled. After the pharmacy interventions, these patients’ blood pressure had significantly improved, with 177 (85.51%) of patients reaching their blood pressure goal (*p* < 0.001). A total of 95 (45.89%) patients were prescribed an appropriate statin at baseline, and after pharmacy interventions 136 (65.7%) patients had met their statin goal, for an increase of 19.8 percentage points (*p* < 0.001).

The average hemoglobin A1c for all the patients at baseline was 9.4%. After the pharmacy clinical interventions, the hemoglobin A1c had decreased by 1.76 percentage points to an average of 7.66% (*p* < 0.001). When reviewing only patients with uncontrolled diabetes (baseline A1c above goal), the hemoglobin A1c average at baseline was 10.35% and had an even larger decrease of 2.23 percentage points to an average of 8.12%. A full analysis of the changes in hemoglobin A1c can be found in Table 2. The average number of anti-diabetic medications the patients were taking at baseline was 1.66, with the majority already having been prescribed metformin (60.2%). After pharmacy intervention, the average number of diabetes medications had only slightly increased to 1.81. The most common addition to a patient’s medication regimen was the increasing use of a GLP-1 agonist. A full breakdown of changes in diabetes medications utilized by drug class can be found in Table 3. 

The average systolic blood pressure (SBP) of patients was reduced from 140 mmHg at baseline to 130.2 mmHg after the CMM visits (*p* < 0.001). A total of 119 patients started off with an uncontrolled blood pressure (BP > 140/90 mmHg), with an average of 152/83 mmHg. After CMM, these patients had a significant improvement in their blood pressure, for a 17 mmHg decrease in SBP and a 6 mmHg decrease in diastolic blood pressure (DBP), for an average blood pressure of 135/77 mmHg. A full description of the blood pressure changes can be found in Table 4. At baseline, the average number of anti-hypertensive medications prescribed per patient was 1.56, and this increased slightly to 1.69 after pharmacy intervention. A description of the changes in antihypertensive medications can be found in Table 5.

Both the ADA and the American College of Cardiology/American Heart Association (ACC/AHA) recommend that all the patients with diabetes between the ages of 40 and 75 years old be prescribed a moderate-to-high intensity statin to decrease the risk of an atherosclerotic cardiovascular disease (ASCVD) event [9,11]. When focusing specifically on the patients between the ages of 40 and 75 years old, about half (51.3%) were on an appropriately dosed statin at baseline. For those between 40 and 75 years old, the results had improved even more, with 72.5% of those patients meeting their statin goal after CMM. In addition to statin therapy, ezetimibe and omega-3 acid ethyl esters were prescribed in a small number of patients. A full list of the changes to lipid-lowering medications can be found in Table 6. 

## 4. Discussion

The data analyzed in this study demonstrates that clinical pharmacists can have positive impacts on patients with diabetes in the primary care setting of a rural healthcare clinic. The improvement in the outcomes of goal attainment rates and decreases in hemoglobin A1c and blood pressure are consistent with findings in other studies [2,3,4,5,6]. The improvement in the primary outcome was statistically and clinically significant. As noted in Figure 1, there is a general shift in improvements in goal attainment after the pharmacist-provided CMM visits. While this primary outcome of goal attainment may be viewed as a surrogate marker for disease control, there are data supporting that achieving controlled glycemic and blood pressure levels with an appropriately dosed statin significantly decreases the risk of long-term complications, including microvascular and macrovascular complications [12,13,14].

The utilization of the progressive collaborative practice agreement is a key element of this CMM service. Without the CPA, the clinical service could not be as efficient, as the pharmacist would need to discuss every recommendation with the physician, which would in turn lead to an increased workload for the physicians. Leveraging this progressive CPA allowed the pharmacists to work at the top of their scope, being readily able to adjust medications based on patient-specific factors.

The data reported in this study represent patient outcomes over three years of this CMM service. While the pharmacists did spend a significant amount of time working with patients to optimize their medication regimens, the patients averaged only 2.23 CMM visits per year. Some patients required only 1 visit per year, while others required up to 11 visits per year. The number of visits needed depended on the severity of the patient’s conditions, the types of medications being used, and the amount of patient counseling that was needed to be provided.

Although there was only a minor increase in the number of anti-diabetic and antihypertensive medications prescribed, the hemoglobin A1c and blood pressure control both improved significantly. This improvement could be attributed to the pharmacist making adjustments to the dosing for the current medications the patient was already taking or switching the patient to an alternative medication, as opposed to simply adding on additional medications. For example, there was an increase in the use of GLP-1 agonists and a decrease in the use of sulfonylureas and prandial insulin. This type of change is consistent with the recent changes to the ADA guideline recommendations for using a GLP-1 agonist as a second-line agent after metformin due to the increasing trials showing the benefits of using this medication class in preventing a cardiovascular event [9]. In addition to medication changes, the pharmacist provided extensive medication and lifestyle education and support throughout the process, which could contribute to the improvements in the A1c and blood pressure lowering without a large increase in medication usage.

In addition to the patient outcomes data presented in this article, there have been many anecdotal benefits of having a pharmacist embedded in a primary care clinic. Other providers, such as attending physicians, medical residents, nurse practitioners, and nurses, have frequently expressed that having a clinical pharmacist as part of the interdisciplinary team is invaluable. Although this clinic has not administered a formal provider satisfaction survey regarding pharmacist-led CMM, other studies in the literature have demonstrated that pharmacists in primary care are well-received by other providers [15,16,17,18]. One study that surveyed 114 primary care providers reported that PCPs believed that the addition of a clinical pharmacist has a highly positive impact on patient care and would highly recommend that other primary care practices integrate a clinical pharmacist. Additionally, that survey reported that 58.78% of respondents believed diabetes was the most valuable disease-focused pharmacy service, and an additional 9.65% of respondents believed it to be hypertension [15]. While the results from that survey cannot be directly applied to this current study, the CMM model has received great feedback from PCPs that highly value and appreciate the service. In addition to providing CMM, the pharmacists in this clinic are also frequently consulted for drug information questions, medication access concerns, or other informal consults. In fact, the success of this CMM service has led to the planned expansion of CMM services to other primary care clinics within this institution.

A limitation of this research analysis is the lack of a patient control group without a clinical pharmacist. Without this control group, it cannot be directly stated that the clinical pharmacy service can improve these patient outcomes to a higher degree than other types of clinicians. Given that this analysis is of patient data from a small rural health clinic, a control group was not logistically possible. Other patients in the clinic who were not seen by the clinical pharmacy team would not be an appropriate comparison, as the patients seen by the clinical pharmacy team are often more complex compared to those solely managed by the PCP. Additionally, the clinic is an interdisciplinary teaching clinic and the majority of PCPs are family medicine resident physicians. The clinical pharmacists frequently provide undocumented and informal consultations with the physicians, so utilizing other patients in the clinic could not be a definite control group. Without the control group, it can still be inferred that the clinical pharmacy service has had positive impacts on patient outcomes as evidenced by the pre- and post-improvements in chronic disease state outcomes. Other studies have included the use of a control group and have reported improved outcomes in the group that includes a pharmacist [6,19,20,21,22,23,24,25]. Although this current study does not include a control group, the improvements in goal attainment and decreases in hemoglobin A1c and blood pressure are consistent with the findings from the other studies. For example, one study conducted at a different institution compared clinics with a pharmacist and clinics without a pharmacist, utilizing the same primary outcome of the composite of goal attainment rates for A1c, blood pressure, and statin therapy. The study concluded that the clinics with the integration of a pharmacist had higher goal attainment rate improvements than the clinics without the pharmacist [6]. Another limitation of this analysis is regarding the dyslipidemia treatment goal. For the purposes of this analysis, the dyslipidemia goal was set as the prescription of a moderate-to-high intensity statin, which is generally recommended for the majority of patients with diabetes aged 40–75 years old. This study did include patients outside of the 40–75 year range and did not assess whether or not a patient had clinical ASCVD or a severely elevated low-density lipoprotein (LDL) at baseline, which are indications for high-intensity statin therapy. The current ACC/AHA guidelines also have added the addition of a secondary LDL goal of <100 mg/dL for patients with diabetes after being prescribed a moderate-to-high intensity or maximally tolerated statin [11]. The LDL levels were not assessed in this data analysis. Additionally, contraindications for statin therapy were not assessed in the data analysis. There may have been reasons why not all the patients were prescribed the statin, including whether they had previously not tolerated a statin medication, had a history of rhabdomyolysis, or had liver dysfunction.

Future areas of interest in this research topic include developing additional methods to analyze a CMM service. Given that pharmacy services in primary care clinics can have widely varying models from different institutions, no formal CMM metric or analysis has become the gold standard. Areas of consideration for future research include CMM effects on patient hospitalization rates, medication adverse effect rates, and quality of life. The outcomes reported in this analysis are focused primarily on the patient outcomes related to diabetes. While this does provide results for the majority of patients managed by this service (61.4% of the total CMM patients were included in this analysis), there were a significant number of patients that did not have diabetes and were managed by the clinical pharmacy service. These patients could have been referred to the pharmacy service for polypharmacy concerns or the management of other non-diabetes chronic conditions such as COPD, heart failure, or anticoagulation management. Given that CMM services provide management for a large range of conditions, it is difficult to determine a single primary outcome to research to assess the entire service. 

This clinical pharmacy service additionally has plans to expand in the future. Currently, the authors are in the process of adding an additional clinical pharmacist to provide a similar CMM service at the other primary care clinics within the network of this institution. This expansion was requested by the medical director who has seen first-hand the added value and improved patient care by integrating a pharmacist. In addition to expanding this model to the other clinics, the authors are considering expanding the service to include a transition of care service. The clinic is located on the same campus as a hospital, where PCPs have inpatient privileges and rotate through the inpatient wards to manage their patients when admitted. This provides a great set-up to have a transition of care service, which would include a patient handoff from acute care clinical pharmacists to the ambulatory care clinical pharmacists for post-discharge management.

## 5. Conclusions

This study has shown that having a clinical pharmacist integrated in a primary care setting has significantly benefited patients with type 1 and type 2 diabetes in obtaining improved control of their condition. The pharmacist’s expertise in CMM management positively impacts patient care, and an expansion of CMM services should be considered.

## Figures and Tables

**Table 1:** Baseline characteristics (N = 207).

CHARACTERISTIC	PATIENT GROUP (N = 207)
MEAN AGE	56.8 years
FEMALE GENDER, %	49.3
AVERAGE A1C, %	9.42
TYPE 2 DIABETES, N (%)	191 (92.27)
AT A1C GOAL, N (%)	50 (24.15)
AVERAGE SBP	140 mmHg
AVERAGE DBP	79.2 mmHg
AT BLOOD PRESSURE GOAL, N (%)	88 (42.51)
AT STATIN GOAL, N (%)	95 (45.89)
0 OF 3 GOALS MET, N (%)	43 (20.8)
1 OF 3 GOALS MET, N (%)	105 (50.7)
2 OF 3 GOALS MET, N (%)	49 (23.7)
3 OF 3 GOALS MET, N (%)	10 (4.8)

**Table 2:** Changes in hemoglobin A1c.

	MEAN A1C BASELINE	MEAN A1C POST CMM	MEAN A1C CHANGE	*p*-Value
ALL PATIENTS (N = 207)	9.42	7.66	−1.76	*p* < 0.001
UNCONTROLLED AT BASELINE (N = 157)	10.35	8.12	−2.23	*p* < 0.001
CONTROLLED AT BASELINE (N = 50)	6.52	6.23	−0.29	*p* = 0.001
TYPE 2 DIABETES (N = 191)	9.27	7.51	−1.77	*p* < 0.001
TYPE 1 DIABETES (N = 16)	11.2	9.54	−1.66	*p* < 0.001
GOAL A1C < 7% (N = 155)	9.4	7.52	−1.88	*p* < 0.001
GOAL A1C < 8% (N = 52)	9.48	8.08	−1.4	*p* < 0.001

**Table 3:** Changes in anti-diabetic medications for patients with type 2 diabetes (N = 191).

MEDICATION TYPE	BASELINE [N, (%)]	POST CMM [N, (%)]	CHANGE [N, (%)]
METFORMIN	115 (60.2)	116 (60.7)	1 (0.5)
SULFONYLUREAS	34 (17.8)	27 (14.1)	−7 (−3.7)
THIAZOLIDINEDIONES	2 (1)	3 (1.6)	1 (0.5)
SODIUM-GLUCOSE TRANSPORT PROTEIN 2 INHIBITORS	3 (1.6)	11 (5.8)	8 (4.2)
DIPEPTIDYL PEPTIDASE 4 INHIBITORS	13 (6.8)	25 (13.1)	12 (6.3)
GLUCAGON-LIKE PEPTIDE-1 AGONISTS	14 (7.3)	41 (21.5)	27 (14.2)
BASAL INSULIN	90 (47)	91 (47.6)	1 (0.5)
PRANDIAL INSULIN	44 (23)	33 (17.3)	−11 (−5.7)

**Table 4:** Changes in blood pressure (BP).

	MEAN BP BASELINE	MEAN BP POST CMM	MEAN CHANGE
ALL PATIENTS (N = 207), SBP	140	130.2	−9.8
ALL PATIENTS (N = 207), DBP	79.2	76	−3.2
UNCONTROLLED AT BASELINE (N = 119), SBP	151.9	134.9	−17
UNCONTROLLED AT BASELINE (N = 119), DBP	83.4	77.2	−6.2
CONTROLLED AT BASELINE (N = 88), SBP	123.9	123.7	−0.2
CONTROLLED AT BASELINE (N = 88), DBP	73.6	74.4	0.8

**Table 5:** Changes in antihypertension medications (N = 207).

MEDICATION TYPE	BASELINE [N, (%)]	POST CMM [N, (%)]	CHANGE [N, (%)]
ACE INHIBITORS	90 (43.3)	84 (40.6)	−6 (−2.7)
ANGIOTENSIN II RECEPTOR BLOCKERS	47 (22.6)	56 (27.1)	9 (4.5)
THIAZIDE DIURETICS	24 (11.5)	33 (15.9)	9 (4.5)
CALCIUM CHANNEL BLOCKERS	44 (21.1)	52 (25.1)	8 (3.9)
BETA BLOCKERS	79 (38)	85 (41.1)	6 (3.1)
OTHERS	35 (16.8)	32 (15.5)	−3 (−1.3)

**Table 6:** Changes in lipid-lowering medications (N = 207).

MEDICATION TYPE	BASELINE [N, (%)]	POST CMM [N, (%)]	CHANGE [N, (%)]
HIGH INTENSITY STATINS	58 (28)	90 (43.5)	32 (15.5)
MODERATE INTENSITY STATINS	38 (18.4)	46 (22.2)	8 (3.8)
LOW INTENSITY STATINS	7 (3.4)	1 (0.5)	−6 (−2.9)
NON-STATINS	8 (3.9)	14 (6.8)	6 (2.9)
